# A clinical trial of super-stable homogeneous lipiodol-nanoICG formulation-guided precise fluorescent laparoscopic hepatocellular carcinoma resection

**DOI:** 10.1186/s12951-022-01467-w

**Published:** 2022-06-03

**Authors:** Pan He, Yongfu Xiong, Jinfa Ye, Biaoqi Chen, Hongwei Cheng, Hao Liu, Yating Zheng, Chengchao Chu, Jingsong Mao, Aizheng Chen, Yang Zhang, Jingdong Li, Jie Tian, Gang Liu

**Affiliations:** 1 State Key Laboratory of Molecular Vaccinology and Molecular Diagnostics, Center for Molecular Imaging and Translational Medicine, School of Public Health, Xiamen University, Xiamen, 361102 China; 2 Department of Hepatobiliary Surgery, Academician (Expert) Workstation, Affiliated Hospital of North Sichuan Medical College, Nanchong, 637600 China; 3 Fujian Provincial Key Laboratory of Biochemical Technology, Institute of Biomaterials and Tissue Engineering, Huaqiao University, Xiamen, 361021 China; 4 Amoy Hopeful Biotechnology Co., Ltd, Xiamen, 361027 China; 5 Key Laboratory of Molecular Imaging, Institute of Automation, Chinese Academy of Sciences, Beijing, 100190 China

**Keywords:** Indocyanine green nanoparticles, Fluorescent laparoscope, Hepatectomy, Translational medicine, Theranostics

## Abstract

**Background:**

Applying traditional fluorescence navigation technologies in hepatocellular carcinoma is severely restricted by high false-positive rates, variable tumor differentiation, and unstable fluorescence performance.

**Results:**

In this study, a green, economical and safe nanomedicine formulation technology was developed to construct carrier-free indocyanine green nanoparticles (nanoICG) with a small uniform size and better fluorescent properties without any molecular structure changes compared to the ICG molecule. Subsequently, nanoICG dispersed into lipiodol *via* a super-stable homogeneous intermixed formulation technology (SHIFT&nanoICG) for transhepatic arterial embolization combined with fluorescent laparoscopic hepatectomy to eliminate the existing shortcomings. A 52-year-old liver cancer patient was recruited for the clinical trial of SHIFT&nanoICG. We demonstrate that SHIFT&nanoICG could accurately identify and mark the lesion with excellent stability, embolism, optical imaging performance, and higher tumor-to-normal tissue ratio, especially in the detection of the microsatellite lesions (0.4 × 0.3 cm), which could not be detected by preoperative imaging, to realize a complete resection of hepatocellular carcinoma under fluorescence laparoscopy in a shorter period (within 2 h) and with less intraoperative blood loss (50 mL).

**Conclusions:**

This simple and effective strategy integrates the diagnosis and treatment of hepatocellular carcinoma, and thus, it has great potential in various clinical applications.

**Supplementary information:**

The online version contains supplementary material available at 10.1186/s12951-022-01467-w.

## Introduction

Hepatocellular carcinoma (HCC) is a common malignancy in humans. According to the latest cancer statistics, HCC is the sixth most common cancer type (among 36 types of cancer), and it is ranked fourth among cancers associated with a high mortality rate [1]. Presently, patients with HCC are treated by radical resection and liver transplantation; however, due to the shortage of liver donors and the high cost of transplantation, radical resection is the primary treatment modality [2]. Regardless, even after radical resection, the 5-year survival rate is less than 5%, and more than 50% of patients will relapse within 3 years [3]. The main reason for this high recurrence rate is that many preoperative imaging methods cannot achieve intraoperative real-time navigation and cannot detect tumors smaller than 1 cm in diameter, leading to incomplete resection of cancer tissues and microsatellite lesions [4, 5]. Therefore, it is essential to develop advanced imaging techniques and multi-functional contrast agents for precise tumor identification and surgical navigation.

To achieve that in clinical practice, it is necessary to accomplish convenient and rapid imaging of lesions. Although computed tomography (CT), magnetic resonance imaging (MRI), positron emission tomography (PET), and contrast-enhanced ultrasonography have been used to diagnose tumors and more precisely identify the boundary between normal and tumor tissues in the clinic [6, 7], these imaging methods possess low spatial resolution or sensitivity, also depend on large instruments that require a professional operation, complex diagnostic interpretations, or associate with radiation injury. Near-infrared fluorescence imaging–with advantages of real-time imaging, non-invasion and high sensitivity–provides a new modality for precise surgical navigation [8, 9]. Indocyanine green (ICG), as the clinical used optical dye, plays a vital role in the localization of tumor lesions and tissue boundaries during surgery [10]. However, ICG is a small molecular substance; its aqueous solution is unstable and easily combined with plasma proteins and is quickly cleared by the liver and excreted out of the body when injected into the body [11]. Furthermore, during real-time intraoperative navigation, due to the aggregation and lack of tumor-specific target of ICG, the fluorescence is easily extinguished, resulting in a false positive rate of up to 40–50%, which seriously restricts the development of fluorescent surgical navigation [12, 13].

Additionally, in clinical treatment, only a small number of patients with liver cancer are suitable for direct surgical resection, and surgery after transcatheter arterial embolization (TAE) is an essential adjuvant technique for patients with advanced HCC. Preoperative adjuvant TAE can clarify the tumor size, tumor extent, tumor number, and vascular variation prior to tumor resection, thereby providing a basis for the choice of surgical approach and resection range. Currently, TAE uses lipiodol as the primary embolic agent, commonly applied in palliative treatment for advanced HCC and adjuvant therapy before hepatectomy. In particular, lipiodol can passively target and accurately deposit in lesions to deliver other theranostic agents to cancer [14]. We developed a super-stable homogeneous intermixed formulation technology (SHIFT), realized the fluorescence-guided surgical navigation that ICG homogeneously intermixed into lipiodol after long-term TAE, and thus constructed a comprehensive treatment plan for liver cancer patients [15, 16]. Furthermore, super-table pure-nanomedicine formulation technology (SPFT), a green physical process, is also developed to construct nano-drugs to improve the properties of molecular drugs, and avoid the drawbacks of other nanofabrication techniques that introduce additional components [17, 18]. In our previous work, ICG nanoparticles (nanoICG) were produced by SPFT to improve the optics and water/thermal stability of the ICG molecule and effectively avoid the decomposition and clearance *in vivo*. After being integrated with lipiodol *via* SHIFT, the nanoICG performed an excellent tumor-specific deposition to visualize the tumor regions for precise resection in VX2 orthotopic hepatocarcinoma models [19].

**Fig. 1:**
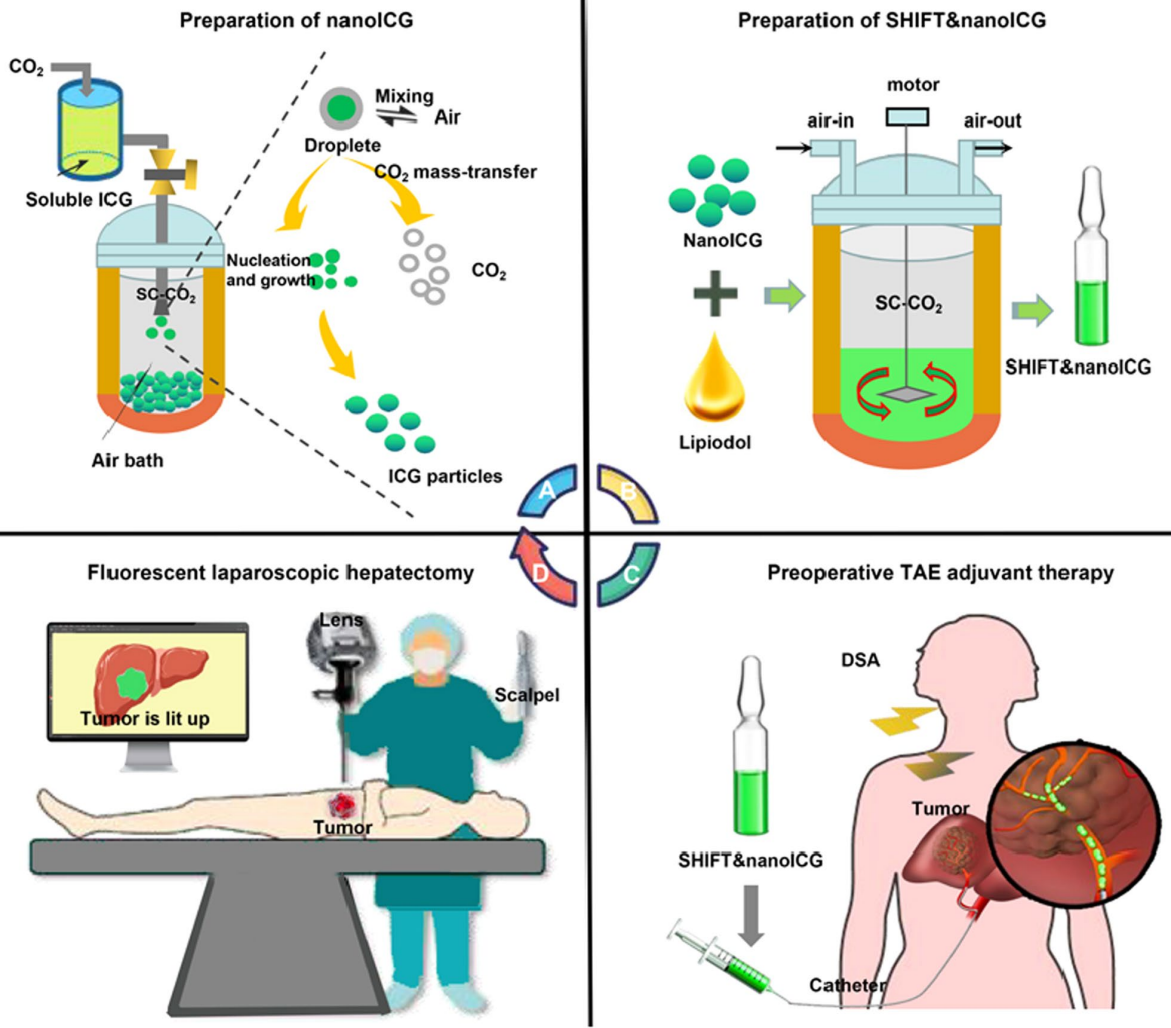
Schematic illustration of research process. **A** Supercritical anti-solvent process was employed to produce carrier-free nanoICG with enhanced imaging properties and anti-photobleaching capacity. **B** Superstable homogeneous intermixed formulation technology (SHIFT) was employed to produce SHIFT&nanoICG for theranostics. **C** Preoperative TAE adjuvant therapy with SHIFT&nanoICG as the embolic agent. **D** The patient received a precise laparoscopic hepatectomy under real-time fluorescence after TAE

This study aimed to determine the specificity, sensitivity, and resection efficiency of SHIFT&nanoICG-assisted surgery for patients with HCC. As shown in Fig. 1, we prepared clinically-used indocyanine green into carrier-free ICG nanoparticles *via* SPFT. We then used SHIFT to produce a homogeneous lipiodol&nanoICG formulation (SHIFT&nanoICG) for precise fluorescent surgical treatment after preoperative TAE. We illustrate that nanoICG shows a homogeneous size, mild-acidic responsiveness, and better fluorescent properties. Especially, nanoICG exhibits excellent resistance to photobleaching, which is nearly 3-times higher than that of ICG molecules. Therefore, the homogeneous SHIFT&nanoICG showed long-term stability, excellent embolization, safety, and efficacy. Subsequently, in the clinical treatment of human HCC, the tumor could be located precisely with excellent fluorescence performance to illuminate the entire lesion and tumor boundary, and even the microsatellite lesion (0.4 × 0.3 cm), which could not be detected by preoperative imaging. In addition, the safety indicators show that SHIFT&nanoICG does not bring obvious safety risks. Therefore, SHIFT&nanoICG is a promising multi-functional formulation for HCC resection with excellent clinical value.

## Materials and methods

### Materials

Indocyanine green was purchased from Dandong Medical and Pharmaceutical Co., Ltd. (Dandong, China). Ethiodized poppyseed oil was purchased from Jiangsu Hengrui Pharmaceutical Co., Ltd. (Jiangsu, China). The anti-Ki-67 antibody, 4′,6-diamidino-2-phenylindole (DAPI), oil red O dye and the terminal deoxynucleotidyl transferase dUTP nick-end labeling (TUNEL) kit were procured from Servicebio (Beijing, China). The SHIFT and SPFT instruments were developed in our lab (patent nos. 2019107349374.4, 201910105683X and 16581600), the Center for Molecular Imaging and Translational Medicine, Xiamen University. The fluorescent surgical navigation system was provided by the Key Laboratory of Molecular Imaging, Institute of Automation, Chinese Academy of Sciences, Beijing and the Beijing Digital Precision Medicine Technology Co., Ltd.

### Preparation and characterization of nanoICG

A total of 50 mg of ICG was dissolved in 10 mL of absolute ethyl alcohol, and the solution was transferred to the supercritical carbon dioxide (SC-CO_2_) reactor. Carbon dioxide (CO_2_) was pumped into the high-pressure vessel at a constant flow rate of 35 g/min to remove the ethyl alcohol, resulting in the precipitation of nanoICG. After the solution was injected into the vessel, CO_2_ was pumped for an additional 20 min to completely remove the ethyl alcohol (Fig. 1A). The nanoICG was characterized by scanning electron microscopy (SEM) using a Leica EM CPD300 scanning electron microscope (Leica Microsystems GmbH, Wetzlar, German) and dynamic light scattering (DLS) using a Malvern zetasizer (Malvern Instruments Ltd., Malvern, UK). The structure of the drug was carried out by liquid chromatography-mass spectrometry (LC-MS) using a Shimadzu high-pressure liquid chromatography (HPLC) system equipped with an SPD-20 A ultraviolet-visible (UV-VIS) light detector (Shimadzu Corp., Kyoto, Japan) Fluorescent reporter detection and fluorescent signal intensity measurements were performed using an IVIS Lumina II fluorescence imaging system (PerkinElmer Inc., Hopkinton, MA, USA).

### Preparation and characterization of SHIFT&nanoICG

A total of 10 mg of nanoICG was dispersed in 10 mL of lipiodol, and the crude lipiodol-nanoICG formulation was transferred to the clean SHIFT reactor [20]. After 1 h, the samples were collected and sterilized by irradiation (Fig. 1B). The viscosities of lipiodol, the crude lipiodol–nanoICG (RL&nanoICG) formulation mixed using manual three-way blending, and SHIFT&nanoICG were detected with a viscosimeter at 37 °C. The radio-imaging properties of lipiodol and SHIFT&nanoICG were investigated using the Siemens Inveon micro-CT scanner (Munich, Germany).

### Determination of cell fluorescence properties and cytotoxicity

LO2, Hepa1-6 and HepG2 cells were seeded in glass-bottom culture dishes at 5 × 10^5^ cells/dish and cultured for 12 h. The freeICG and nanoICG (ICG, 20 µg/mL) were added to the cell culture medium, and the plates were incubated for 6 h in the dark. Next, the cell culture medium was removed, and the cells were washed with phosphate buffered saline (PBS). The nuclei were stained with 4-amino-6-diamino-2-phenylindole (DAPI), and fluorescent images of the cells were obtained using a Leica confocal microscope and a LAS X confocal scanning system and fluorescence intensity was measured using Image J software.

The cytotoxicity of freeICG and nanoICG was examined by the Cell Counting Kit-8 (CCK-8) assay. LO2, Hepa1-6 and HepG2 cells were seeded in 96-well plates at 1 × 10^4^ cells/well and incubated for 12 h. The freeICG and nanoICG (ICG, 12.5, 25, 50 and 100 µg/mL) were added to the cell culture medium, and the plates were incubated for 24 h. The CCK-8 assay was performed according to the manufacturer’s instructions, and the cytotoxicity of freeICG and nanoICG was determined.

### Preoperative adjuvant TAE treatment

Embolism and hepatectomy in a clinical case of HCC involving human participants were carried out in accordance with the ethical standards of the institutional and/or national research committee and the 1964 Helsinki Declaration and its later amendments or comparable ethical standards. The study was approved by the Institutional Review Board of Affiliated Hospital of North Sichuan Medical College (IRB No. 2022ER146-1) and registered at chictr.org.cn (ID: ChiCTR2200058803). The subjects signed and gave the written informed consent.

Using the Seldinger technique, the right femoral artery was punctured and intubated into the common hepatic artery. After the injection of contrast medium, digital subtraction angiography (DSA) was performed to check the blood supply of the hepatic artery, as well as the size, number and location of the intrahepatic tumor. Next, according to the tumor size, a pre-defined amount of SHIFT&nanoICG was injected to embolize the tumor-feeding artery until target artery blood stasis or regurgitation was achieved (Fig. 1C).

### Fluorescent laparoscopic hepatectomy

After intubation and general anesthesia, the head was slightly raised in the supine position, and a small incision (length, 10 mm) was made at the inferior margin of the umbilical cord to establish CO_2_ pneumoperitoneum (12–14 mmHg) and then a laparoscope was inserted. Under the guidance of the laparoscope, a trocar (diameter, 12 mm) was placed on the outer edge of the right rectus abdominis muscle and under the costal edge of the left anterior axillary line, while another trocar (diameter, 5 mm) was placed on the outer edge of the superior left rectus abdominis muscle and under the costal edge of the right anterior axillary line. To fully expose the liver, an electric knife or ultrasound knife was used to dissociate the peri-hepatic ligament and surrounding tissue.

Before tumor resection, the fluorescent laparoscopic system (FLS) was used to observe the suspicious sites from a distance of 10–40 cm. After tumor resection, the FLS was used to examine whether there was a fluorescent signal at the resected site or in the harvested specimen (Fig. 1D). All specimens were submitted for pathological analysis. Meanwhile to evaluate the SHIFT&nanoICG fluorescent navigation effect, the tumor-to-normal tissue ratio were calculated using Image J and regions of interest (ROIs).

### Histology and immunohistochemistry

The tissues were fixed in 45% paraformaldehyde and embedded in paraffin, followed by staining with hematoxylin eosin (H&E), oil red O, an anti-Ki-67 antibody, TUNEL and/or DAPI. All tissue sections were examined by light or fluorescence microscopy. Tumor tissues were characterized by the shape of epithelial cells and the pattern of trabecular growth [21]. All tissue sections were analyzed by researchers with at least 10 years of pathology experience.

### Statistical analysis

The data are presented as mean ± standard deviation (SD). Each experiment was performed three independent times. *P*-values < 0.05 were considered statistically significant (*P < 0.05; **P < 0.01; ***P < 0.001; ****P < 0.0001).

## Results and discussion

### Preparation and characterization of nanoICG

**Fig. 2:**
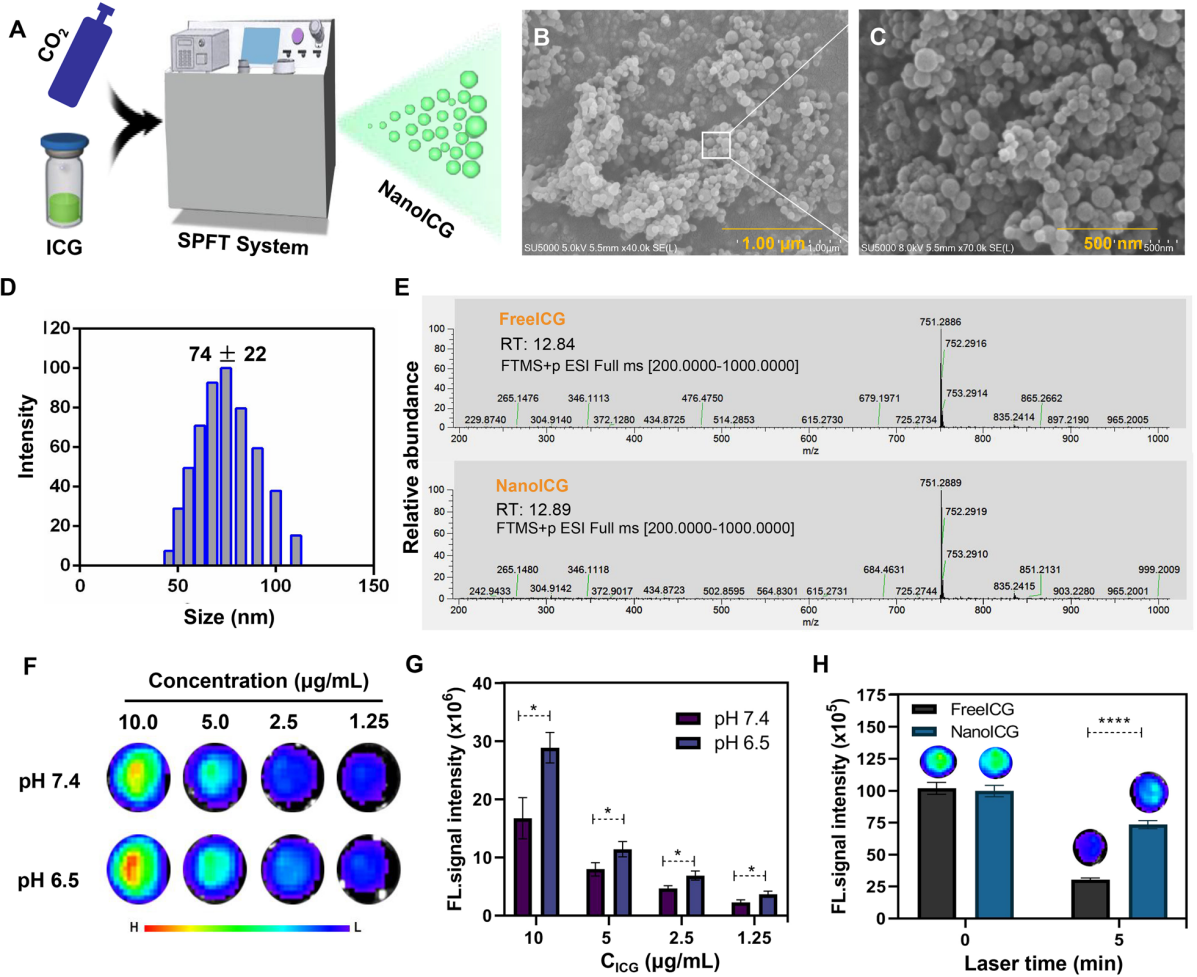
Preparation and characterization of nanoICG. **A** Schematic of nanoICG preparation *via* SPFT for clinical diagnosis and treatment. **B**, **C** mixture due to the pelleted Representative SEM images of nanoICG, scale bar: 1 μm (**B**) and 500 nm (**C**). **D** DLS of nanoICG. E The molecule structure of LC-MS of freeICG and nanoICG. **F**, **G** The fluorescence intensities of nanoICG in different pH conditions were detected by IVIS Lunima LT at the 745 nm excitation after 1 h incubation. **H** Fluorescence intensity of freeICG and nanoICG was detected at different times by a 745 nm excitation when pH is 6.5. Data represent mean ± SD, n = 3. * *P* < 0.05, **** *P* < 0.0001, Student’s t-test

In terms of tumor imaging and diagnosis, several advances have been made in the ICG nanoparticle field, including inorganic and polymer nanoparticles. ICG nanoparticles show excellent fluorescence intensity and tissue penetration, but their preparation is a complex and limited application [22, 23]. Herein, we describe a simple, green, and economical approach involving a supercritical CO_2_ anti-solvent process in customizing ICG nanoparticles (Fig. 2A). In the preparation of nanoICG, no organic solvents or toxic reagents were introduced. The SEM results confirmed that nanoICG was successfully prepared as spherical particles (Fig. 2B, C), and the results of DLS showed that the hydrate particle size of nano-ICG was 74 ± 22 nm (Fig. 2D) with a low particle dispersion index (PDI) of 0.066. The results of LC-MS revealed the molecular structure of nanoICG to be similar to that of freeICG (Fig. 2E), indicating that SPFT did not produce structural changes and that there were no other additives. Therefore, nanoICG should have outstanding safety.

Excellent fluorescence performance is critical for successful fluorescence navigation surgery. Previous studies have reported that nano-crystallization can enhance the fluorescence intensity and photostability of ICG [24, 25]. The fluorescence imaging results demonstrated that nanoICG exhibited an excellent fluorescence intensity and even a pH dependence. The fluorescence intensity was enhanced in a mild-acid solution (pH 6.5) relative to a neutral solution (Fig. 2F, G). Additionally, nanoICG displayed outstanding anti-photobleaching capability, enabling much higher photostability than freeICG (Fig. 2H). These findings indicate that nanoICG has excellent clinical application value.

### Preparation and characterization of SHIFT&nanoICG

**Fig. 3:**
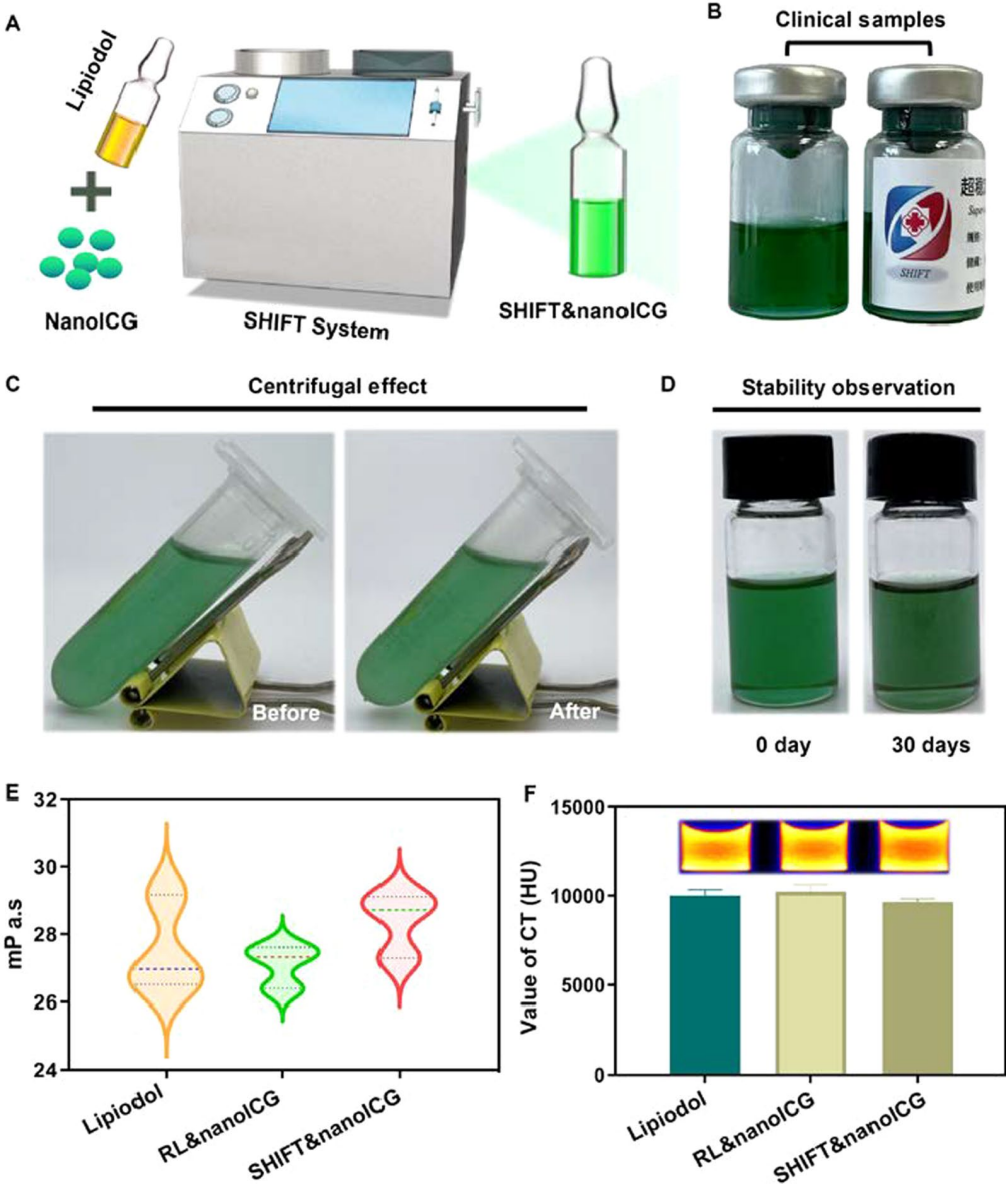
Preparation and characterization of SHIFT&nanoICG.** A** Schematic of SHIFT&nanoICG preparation *via* SHIFT for clinical diagnosis and treatment. **B** Clinical drug samples prepared for TAE treatment. **C** Photograph of the centrifuged formulation. **D** Photograph of SHIFT&nanoICG freshly prepared and stored for 30 days. **E** Viscosity of lipiodol, RL&nanoICG, and SHIFT&nanoICG. **F** CT capacities of lipiodol, RL&nanoICG, and SHIFT&nanoICG

To seamlessly integrate the diagnosis and treatment of HCC by combining the advantages of preoperative TAE and nanoICG, a critical problem had to be solved: how to realize the long-term, stable, and uniform dispersion of amphiphilic ICG in lipiodol. In previous study, we constructed a super-stable, homogeneous and intermixed formulation technology in which the drug molecule could be homogeneously dispersed in lipiodol [15, 18, 19]. The SHIFT&nanoICG formulation prepared by SHIFT showed excellent dispersibility and stability (Fig. 3A, B). To further verify the stability of the formulation, we centrifuged SHIFT&nanoICG at 5000 rpm for 5 min. After centrifugation, nanoICG remained uniformly and steadily dispersed in lipiodol (Fig. 3C). After SHIFT&nanoICG was stored at 25 °C for 30 days, SHIFT&nanoICG was still a transparent and uniformly green suspension, with no apparent stratification or precipitation (Fig. 3D).

The performance of CT imaging and the viscosity of lipiodol is critical to ensuring the safe implementation and embolization of TAE. Effective CT performance can help the operator determine the injection location and dose of lipiodol. The appropriate viscosity is the prerequisite to ensuring good tumor deposition to prevent ectopic embolization [26]. The results showed that this technique’s viscosity and CT value of SHIFT&nanoICG prepared were not different from those of RL&nanoICG and simple lipiodol (Fig. 3E, F), which effectively prevents the risk of ectopic embolism caused by rupture of the tumor-feeding artery due to excessive injection doses.

### Cellular fluorescence properties and cytotoxicity of nanoICG

**Fig. 4:**
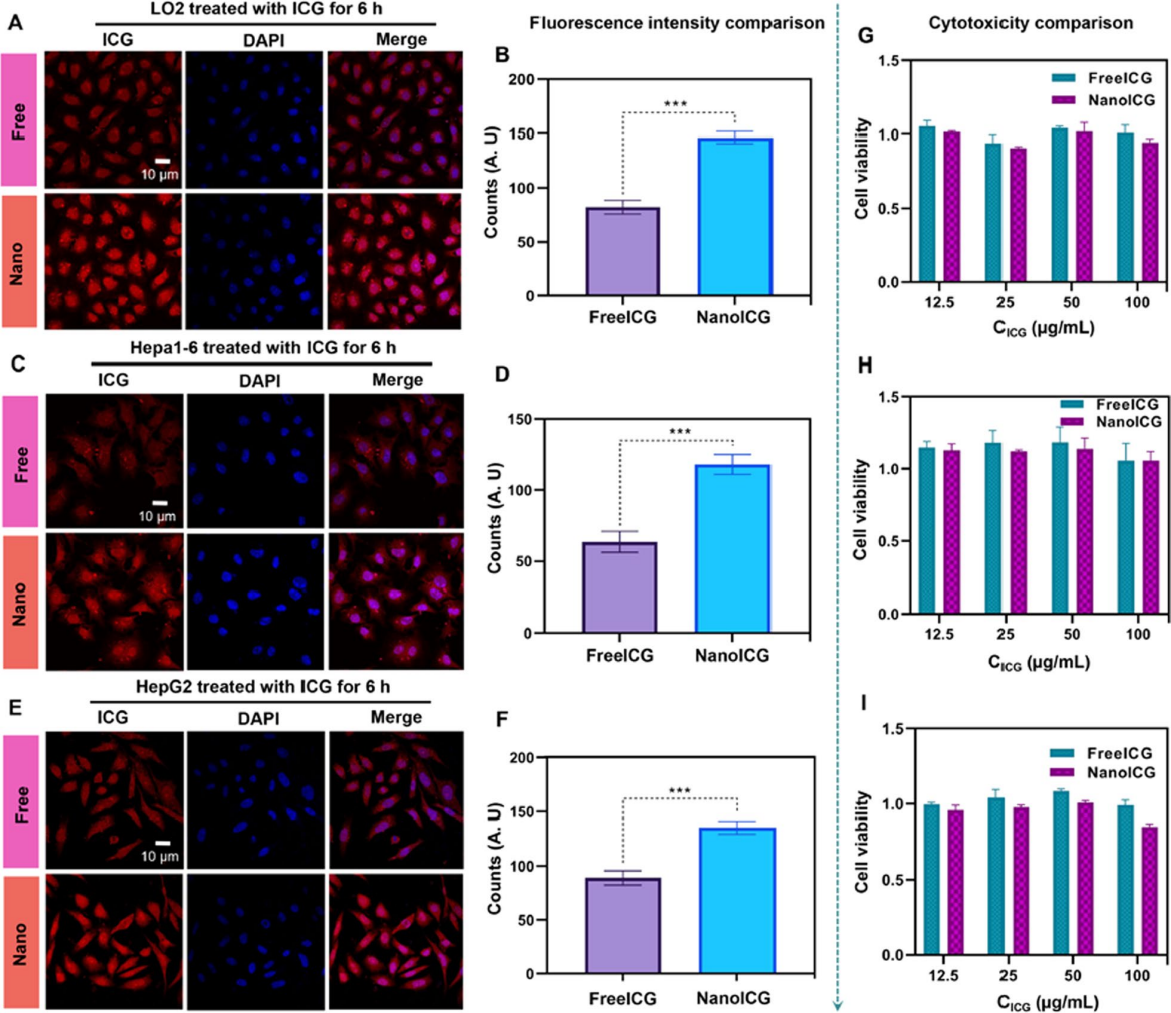
Cellular fluorescence properties and cytotoxicity.** A, B** The fluorescence study of the freeICG/nanoICG in LO2 cells. **C, D** The fluorescence study of the freeICG/nanoICG in Hepa1-6 cells. **E, F** The fluorescence study of the freeICG/nanoICG in HepG2 cells. **G–I** CCK-8 of the freeICG and nanoICG in LO2 (G), Hepa1-6 (H) and HepG2 (I) cells. Data represent mean ± SD, n = 3. *** p < 0.001, Student’s t-test

In fluorescence navigation surgery, the fluorescence performance of ICG determines the quality of the fluorescence navigation surgery [27]. To confirm the imaging ability of nanoICG, we imaged normal liver cell line (LO2) and liver cancer cell lines (Hepa1-6 and HepG2) cells. The results all showed that, compared with freeICG, nanoICG exhibited better cell fluorescence, and the fluorescence intensity increased with the increase in the concentration of nanoICG (Fig. 4A–F), which was indicative of the good cellular internalization of nanoICG, suggesting that the nano-morphology was more permeable [28]. Furthermore, the cytotoxicity of nanoICG was evaluated by the CCK-8 assay, and the nanoICG prepared by SPFT showed no cytotoxicity in LO2 (Fig. 4G), Hepa1-6 (Fig. 4 H), and HepG2 (Fig. 4I) cells at 24 h, even at a concentration of 100 µg/mL, and no significant difference compared with free ICG, revealing that nanoICG is as safe as freeICG.

### Embolism and safety evaluation in a clinical case of HCC

**Fig. 5:**
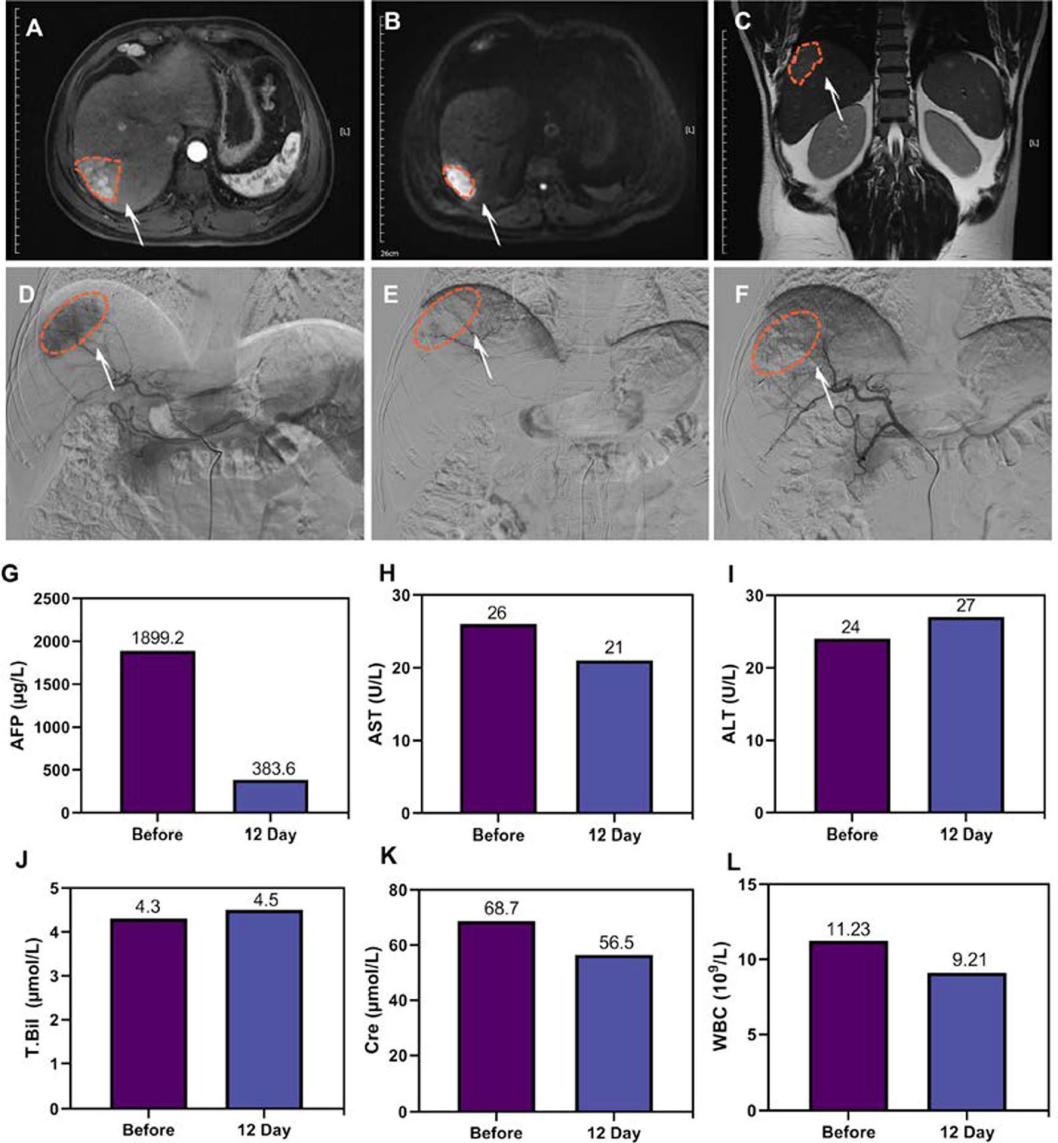
Embolism and safety evaluation in clinical case of HCC.** A–C** MRI showed that a liver tumor at the right posterior lobe with a maximum diameter of 4.3 cm was observed (arrow): 4 × 2.4 × 4.3 cm^3^ (A: arterial phase, B: venous phase, C: coronal). **D–F** DSA angiography and SHIFT&ICG embolization (D: before embolization, E: during embolization, F: after embolization). **G** Blood AFP value (reference interval: 0.0 ~ 20.0 µg/L) before and 12 days after TAE. **H–L** Blood AST (reference interval: 15.0 ~ 40.0 U/L), ALT (reference interval: 9.0 ~ 50.0 U/L), T. Bil (reference interval: 1.7 ~ 26.0 µmol/L), Cre (reference interval: 57.0 ~ 97.0 µmol/L) and WBC value (reference interval: 3.5 ~ 9.5 10E9/L) before and 12 days after TAE

A 52-year-old middle-aged man was admitted to the hospital because of a liver mass found by routine physical examination. On admission, contrast-enhanced magnetic resonance imaging showed a solitary liver tumor (4 × 2.4 × 4.3 cm^3^) in the right posterior lobe without metastatic lesions and satellite lesions (Fig. 5A–C). There was no history of past diseases. After communicating with the patient and informing him of the condition and the follow-up treatment plan, the patient opted for preoperative TAE (SHIFT&nanoICG) assisted fluorescence laparoscopic hepatectomy and signed the consent form. Subsequently, TAE with SHIFT&nanoICG combined with fluorescence laparoscopic hepatectomy was performed. All procedures involving human participants were carried out in accordance with the ethical standards of the institutional and national research committee and the 1964 Helsinki Declaration and its later amendments or comparable ethical standards. All participants received approval from their local institutional review board.

In cases of HCC, 90–95% of the blood is supplied by the hepatic artery, and the embolization of tumor-supplying arteries is common [29]. The key advantage of this adjuvant technique is that TAE can block the tumor-feeding artery to induce necrosis, reduce the tumor size, form a capsule, and reduce the risks of intraoperative bleeding and metastasis. It can also be used in conjunction with hepatic arteriography, tumor-feeding artery selection and passive targeting to detect and eliminate micrometastases and portal vein tumor thrombi to guide radical resection and reduce postoperative tumor recurrence [30]. More importantly, it can passively target and accurately deposit SHIFT&nanoICG embolic agents with therapeutic effects and fluorescent properties within lesions to specifically treat tumors and achieve subsequent precise fluorescence navigation [14]. In this study, during the implementation of TAE, DSA imaging could detect the primary lesion (right posterior lobe) and blood supply (Fig. 5D), and SHIFT&nanoICG could be effectively deposited within the primary lesion (Fig. 5E, F). Tumor markers were re-examined 12 days after surgery, and the alpha-fetoprotein level decreased from 1889.2 to 383.6 µg/L (Fig. 5G), indicating that SHIFT&nanoICG can effectively embolize tumor blood vessels and inhibit tumor development. Moreover, serum biochemical analysis at 12 days after TAE showed no differences in the patient’s white blood cell (WBC) count, as well as the levels of alanine transaminase (ALT), aspartate transaminase (AST), total bilirubin (T. Bil) and creatinine (Cre), compared with before surgery (Fig. 5H–L). In addition, no differences were observed in coagulation indicators, namely, prothrombin times (PT), activated partial thromboplastin time (APTT), and fibrinogen and fibrin degradation products (FDP), before and after surgery (Additional file 1: Fig. S1–3), suggesting that SHIFT&nanoICG are safe and effective.

### Effectiveness of fluorescence-guided hepatectomy after TAE-assisted therapy

**Fig. 6:**
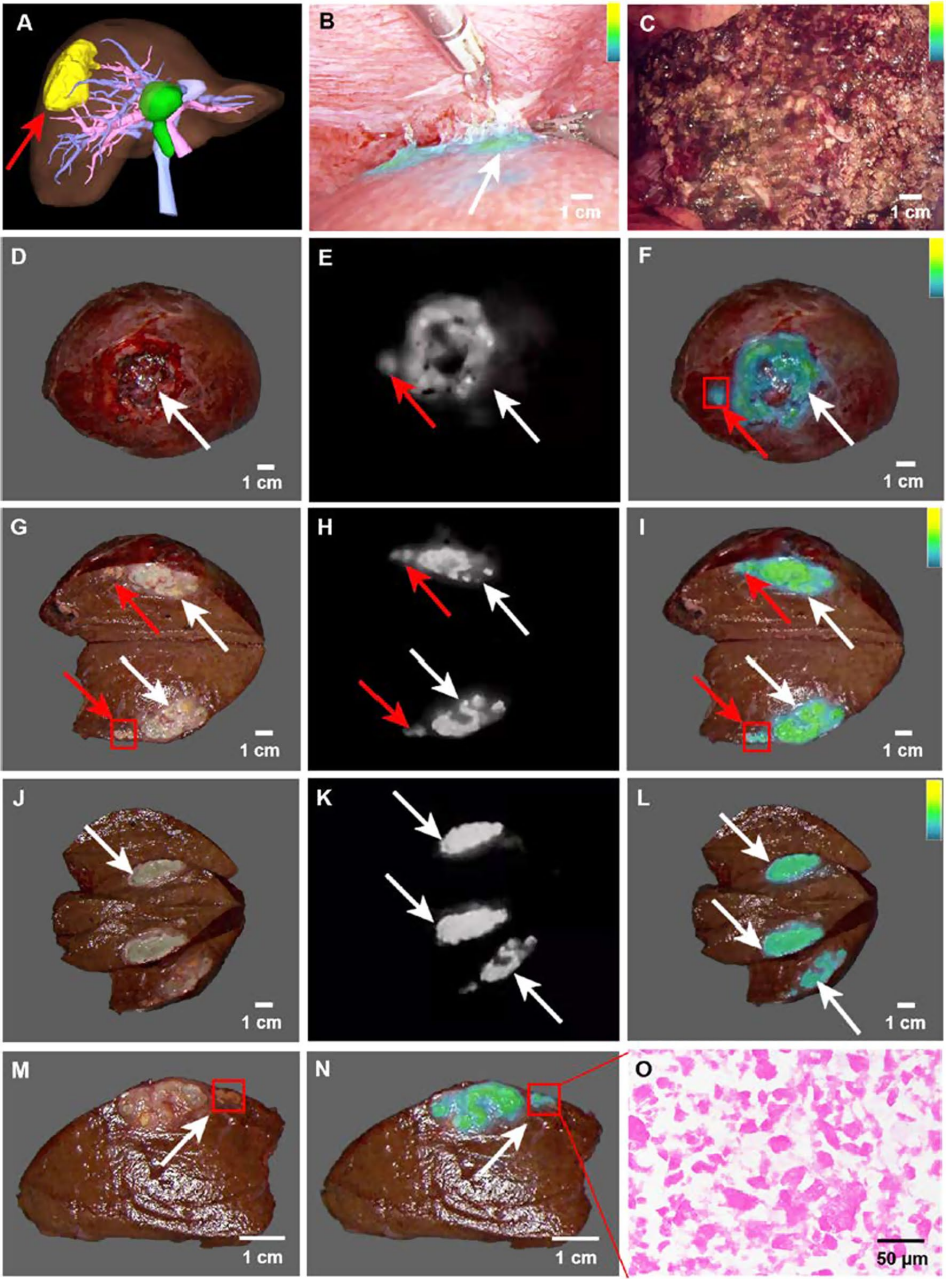
The surgical navigation effect of SHIFT&nanoICG after TAE-assisted therapy. **A** The 3D reconstruction imaging before hepatectomy showed that the tumor was located in the right posterior lobe of the liver and was closely connected to the surrounding blood vessels. **B** The fluorescence imaging of primary lesion. **C** The fluorescence imaging of the incisal margin of the residual liver, with achieved R0 resection. **D–F** Whole resected tumor lesions and fluorescence imaging (white arrowhead = primary lesion, red arrowhead = microsatellite lesion). **G–I** The dissected tumor lesion and fluorescence imaging (white arrowhead = primary lesion, red arrowhead = microsatellite lesion). **J–L** The dissected tumor lesion by layer and fluorescence imaging. **M–N** The microsatellite lesion and fluorescence imaging. **O** The H&E staining of microsatellite lesion

Innovative technologies are critical for driving medical advancements. To confirm the clinical value of the nanoICG-based SHIFT lipiodol preparation in follow-up fluorescence-guided surgical resection, fluorescent laparoscopic hepatectomy was performed on the patient after 2 weeks when the local inflammatory reaction had subsided. Preoperative 3D reconstruction imaging showed that the tumor was located in the right posterior lobe of the liver and was closely connected to the surrounding blood vessels (Fig. 6A). The tumor was located behind the liver, indicating that it was challenging to locate it with traditional methods due to the obstruction of the right anterior lobe of the liver. With SHIFT&nanoICG fluorescence navigation, the location and boundary of the primary tumor were located quickly and precisely, and complete resection of the tumor tissue was completed in a shorter period (within 2 h) and with less intraoperative blood loss (50 mL) (Fig. 6B). Postoperative fluorescence (Fig. 6C) and CT (Additional file 1: Fig. S4) scan imaging of residual liver showed no fluorescence, lipiodol, or residual cancer tissue, which enhanced the reliability of complete R0 resection.

To clarify the effect and value of SHIFT&nanoICG in imaging tumors, we examined the postoperative tumor specimens and observed that tumor lesions were surrounded by SHIFT&nanoICG fluorescence (Fig. 6D–F, white arrow). Furthermore, the tumor boundary could be detected with an excellent signal-to-noise ratio (mean = 1.79) (Additional file 1: Fig. S5A) and microsatellite lesions, which preoperative MRI and 3D images could not detect, were well illuminated (Fig. 6D–F, red arrow). These findings indicate that this technique can lower the incidence of metastasis [31, 32]. When the lesions were incised, the tumor boundary (Fig. 6G–I, white arrow) and microsatellite lesions (Fig. 6G–I, red arrow) marked by SHIFT&nanoICG fluorescence were visible (Additional file 2: Video S1) and had excellent TNR (mean = 2.11) (Additional file 1: Fig. S5B). Astonishing fluorescence performance, accuracy, and TNR (mean = 2.04) were maintained when the tumor was dissected layer by layer (Fig. 6J–L, S5C, Additional file 3: Video S2). After surgery, H&E staining of microsatellite foci indicated that HCC further enhanced our excellent fluorescence performance results (Fig. 6M–O). This excellent and accurate fluorescence performance was a result of the excellent photobleaching resistance, enhanced permeability, the retention effect of nanoICG, the excellent stability and uniformity of SHIFT&nanoICG, and the accurate passive targeting of TAE [15, 19, 30, 33]. In particular, the trace amount of SHIFT&nanoICG deposited in the normal liver tissue around the tumor was metabolized 2 weeks after TAE [34].

In addition, we pathologically analyzed the postoperative tumor specimens. The results of H&E staining revealed a moderately differentiated hepatocellular carcinoma with extensive necrosis (Additional file 1: Fig. S6A). Oil red staining indicated that a large amount of lipiodol was deposited within the lesion (Additional file 1: Fig. S6B), and bright fluorescence was emitted under laser excitation (Additional file 1: Fig. S6C), suggesting that this technique is not affected by the differentiation degree of HCC and is more effective than traditional fluorescence navigation techniques that cannot detect moderately and poorly differentiated HCC [10]. The results of immunohistochemistry showed weak Ki-67 expression and a large number of TUNEL expression in lesions (Additional file 1: Fig. S6D), suggesting that a large number of tumor cells were necrotic after TAE treatment, which could effectively prevent the risk of cancer cell expansion caused by squeezing during the operation [35] and realize the integration of diagnosis and treatment.

## Conclusion

In summary, SHIFT&nanoICG possesses the excellent anti-photobleaching ability, imaging sensitivity of pure nanoICG and deposition of lipiodol within tumors, thereby addressing the clinical issues of fluorescent surgical navigation to realize fluorescent-guided hepatectomy, even for microsatellite lesions. As such, it has great potential for use in clinical settings.

## Supplementary Information

**Additional file 1: Fig. S1.** Blood PT value (reference interval: 10.00 ~ 14.00 s) before and 12 days after TAE.  **Fig. S2 **Blood APPT value (reference interval: 24.00 ~ 39.00 s) before and 12 days after TAE. **Fig. S3. **Blood FDP value (reference interval: 0.00 ~5.00 Ug/mL) before and 12 days after TAE. **Fig. S4 **The CT imaging after fluorescent laparoscopic hepatectomy. **Fig. S5 **The TNR of whole resected tumor lesion (A), dissected tumor lesion (B), dissected tumor lesion by layer (C). **Fig. S6 **Histopathological examination. **A-C **The H&E, oil red staining, and fluorescencesignal of the primary tumor lesion in this patient. **D** The immunofluorescence histological analysis of the primary tumorlesion in this patient showed that there was a low expression rate of Ki-67, and a high expression rate of TUNEL.

**Additional file 2: Vedio S1.** Vedio of the fluorescent imaging effect when the tumor was dissected.

**Additional file 3: Vedio S2.** Vedio of the fluorescent imaging effect when the tumor was dissected by layer.

## Data Availability

All data generated in this study are included in this publication.
