# Supplementary material for: A clinical trial of super-stable homogeneous lipiodol-nanoICG formulation-guided precise fluorescent laparoscopic hepatocellular carcinoma resection
Source: J Nanobiotechnology. 2022 Jun 3;20:250. doi: 10.1186/s12951-022-01467-w (PMC9164554; doi:10.1186/s12951-022-01467-w)
Supplement: Supplementary file 1 — Additional file 1: Fig. S1. Blood PT value (reference interval: 10.00 ~ 14.00 s) before and 12 days after TAE. Fig. S2 Blood APPT value (reference interval: 24.00 ~ 39.00 s) before and 12 days after TAE. Fig. S3. Blood FDP value (reference interval: 0.00 ~5.00 Ug/mL) before and 12 days after TAE. Fig. S4 The CT imaging after fluorescent laparoscopic hepatectomy. Fig. S5 The TNR of whole resected tumor lesion (A), dissected tumor lesion (B), dissected tumor lesion by layer (C). Fig. S6 Histopathological examination. A-C The H&E, oil red staining, and fluorescencesignal of the primary tumor lesion in this patient. D The immunofluorescence histological analysis of the primary tumorlesion in this patient showed that there was a low expression rate of Ki-67, and a high expression rate of TUNEL. [file 12951_2022_1467_MOESM1_ESM.doc]

**Supplementary Information**

**A clinical trial of super-stable homogeneous lipiodol-nanoICG formulation- guided precise fluorescent laparoscopic hepatocellular carcinoma resection**

**He et al.**

**Table of Contents:**

**-Supplementary Figures 1-6**

**A clinical trial of super-stable homogeneous lipiodol-nanoICG formulation- guided precise fluorescent laparoscopic hepatocellular carcinoma resection**

Pan He ^1, #^, Yongfu Xiong ^1, 2,#^, Jingfa Ye ^1, #^, Biaoqi Chen ^3^, Hongwei Cheng ^1^, Hao Liu ^3^, Yating Zheng ^1^, Chengchao Chu ^1, 4^, Jingsong Mao ^1^, Aizheng Chen ^3^, Yang Zhang ^1^, *, Jingdong Li ^2^, *, Jie Tian ^5^, *, Gang Liu ^1^, *

^1^ State Key Laboratory of Molecular Vaccinology and Molecular Diagnostics, Center for Molecular Imaging and Translational Medicine, School of Public Health, Xiamen University, Xiamen 361102, China.

^2^ Department of Hepatobiliary Surgery, Academician (Expert) Workstation, Affiliated Hospital of North Sichuan Medical College, Nanchong 637600, China.

^3^ Fujian Provincial Key Laboratory of Biochemical Technology, Institute of Biomaterials and Tissue Engineering, Huaqiao University, Xiamen 361021, China.

^4^ Amoy Hopeful Biotechnology Co., Ltd., Xiamen 361027, China.

^5^ Key Laboratory of Molecular Imaging, Institute of Automation, Chinese Academy of Sciences, Beijing 100190, China.

* Corresponding author. E-mail addresses: zhangyang0823@xmu.edu.cn (Y. Zhang); Li-Jingdong@hotmail.com (J. Li); [tian@ieee.org](mailto:tian@ieee.org) (J. Tian); gangliu.cmitm@xmu.edu. [cn](mailto:gangliu.cmitm@xmu.edu.cn) (G. Liu).

# P. He, Y. Xiong, and J. Ye contributed equally to this work.

**
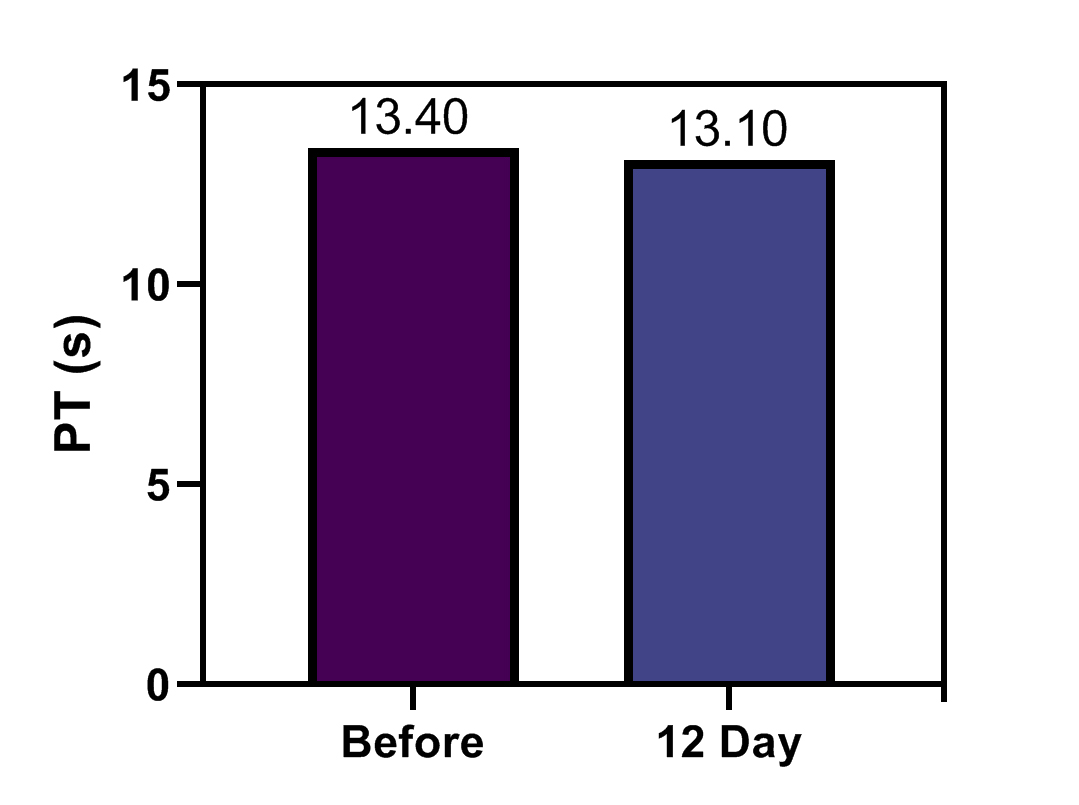
**

**Fig. S1** Blood PT value (reference interval: 10.00 ~ 14.00 s) before and 12 days after TAE.


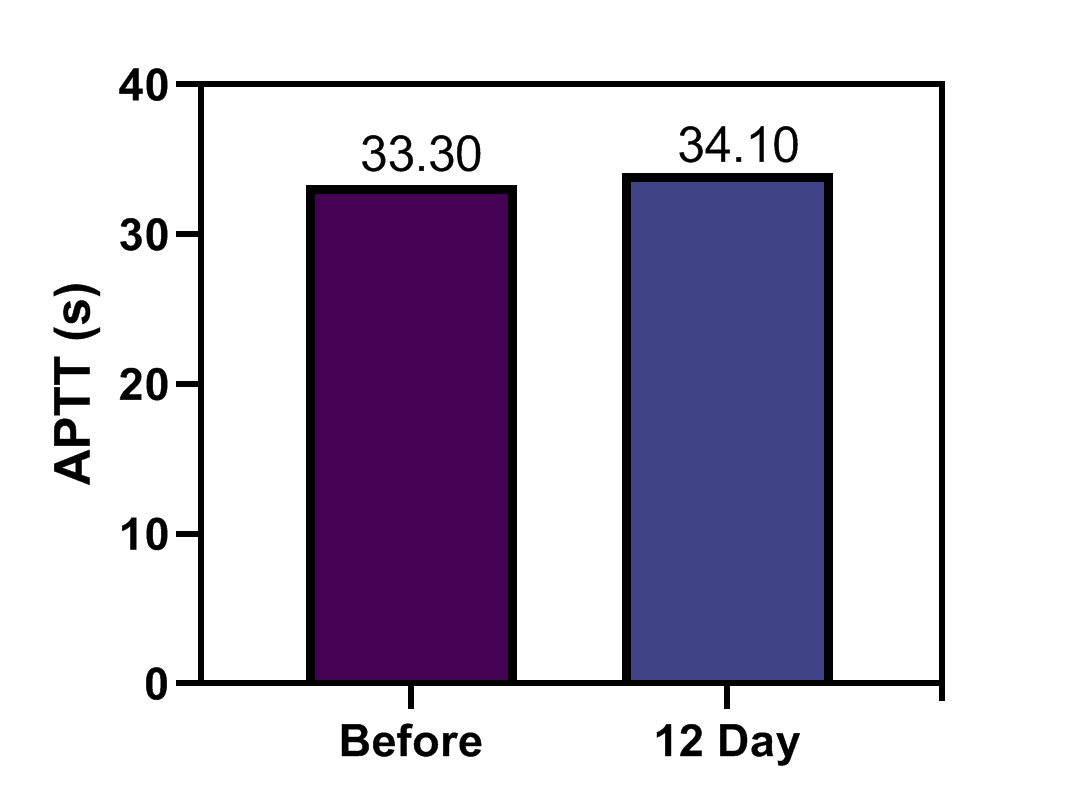


**Fig. S2** Blood APPT value (reference interval: 24.00 ~ 39.00 s) before and 12 days after TAE.


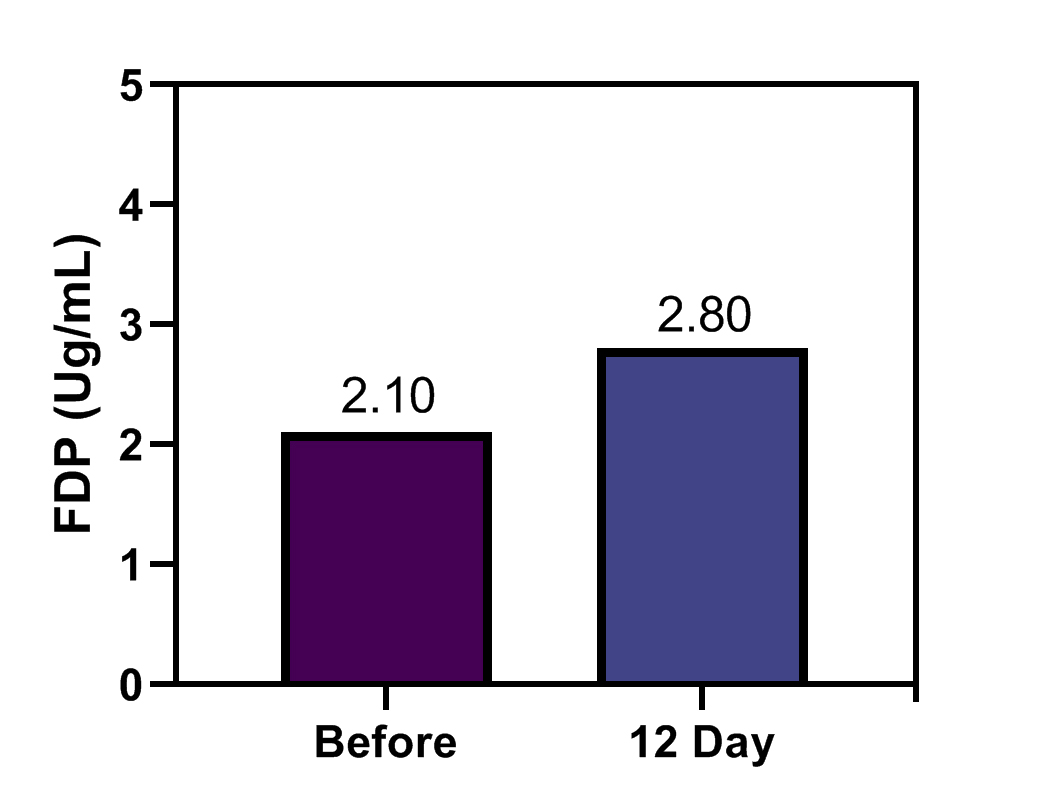


**Fig. S3** Blood FDP value (reference interval: 0.00 ~ 5.00 Ug/mL) before and 12 days after TAE.


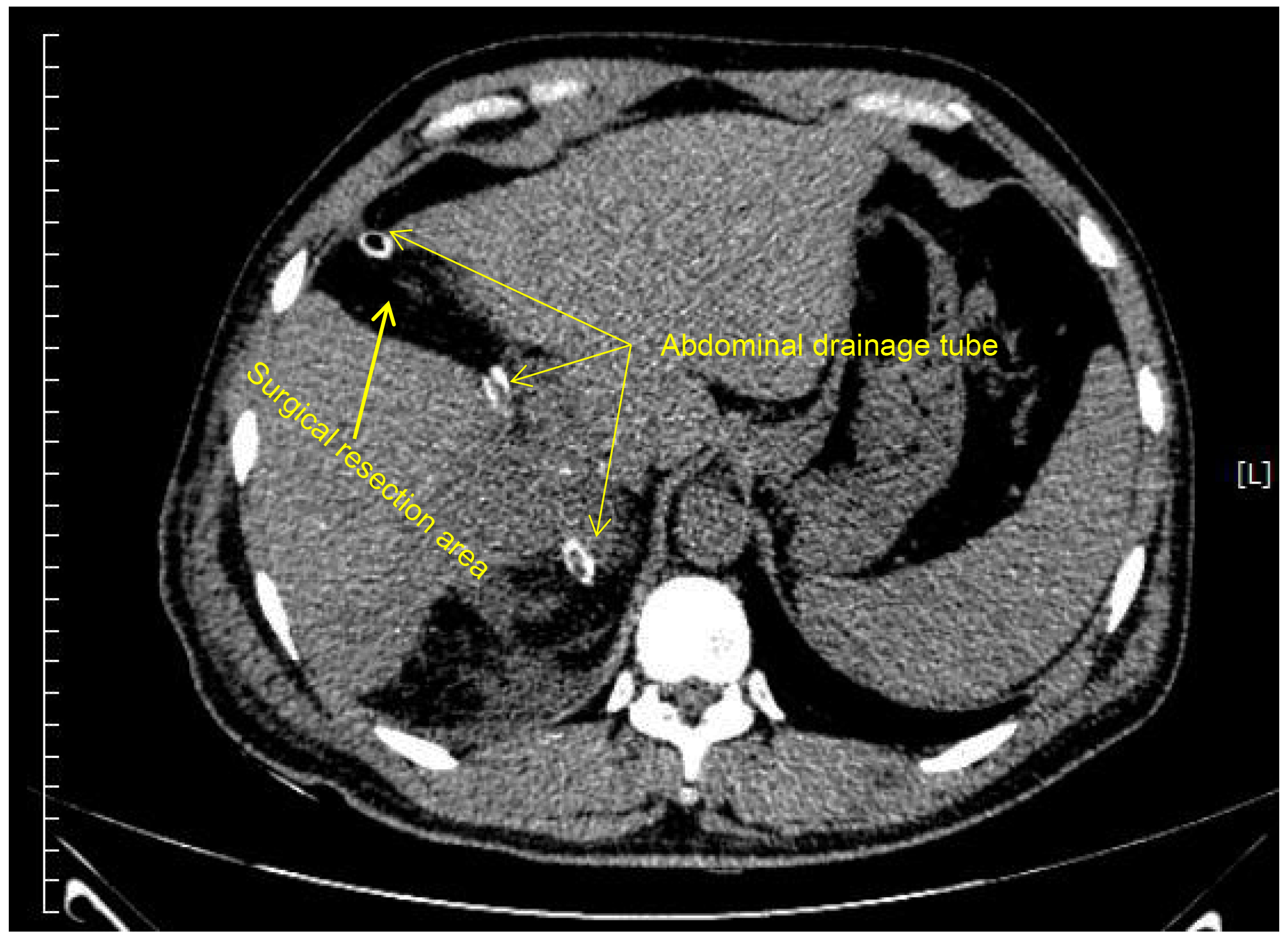


**Fig. S4** The CT imaging after fluorescent laparoscopic hepatectomy.


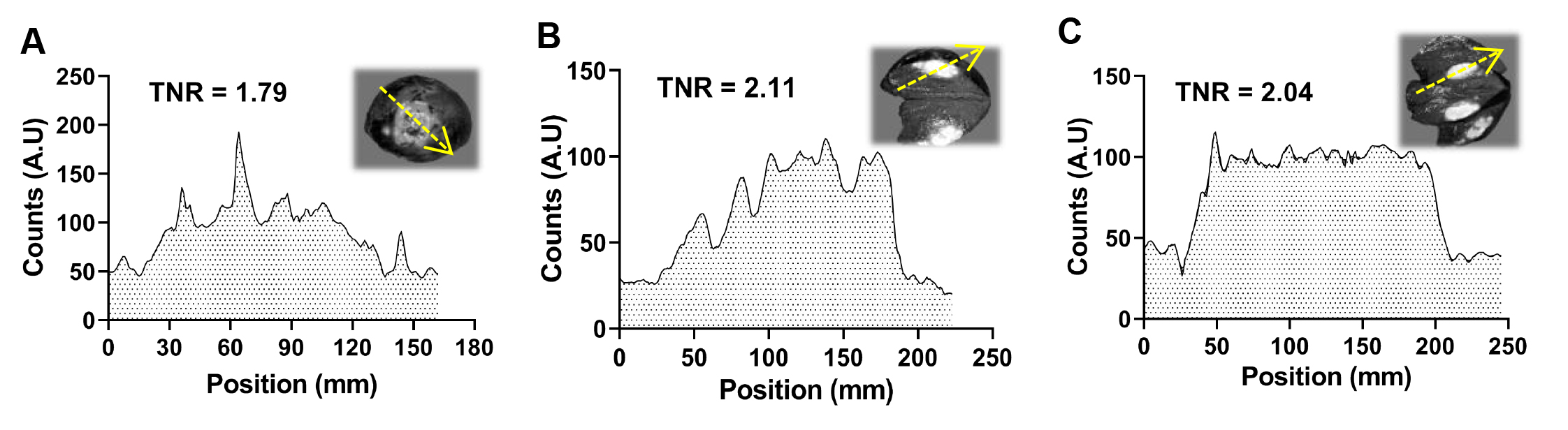


**Fig. S5** The TNR of whole resected tumor lesion (A), dissected tumor lesion (B), dissected tumor lesion by layer (C).


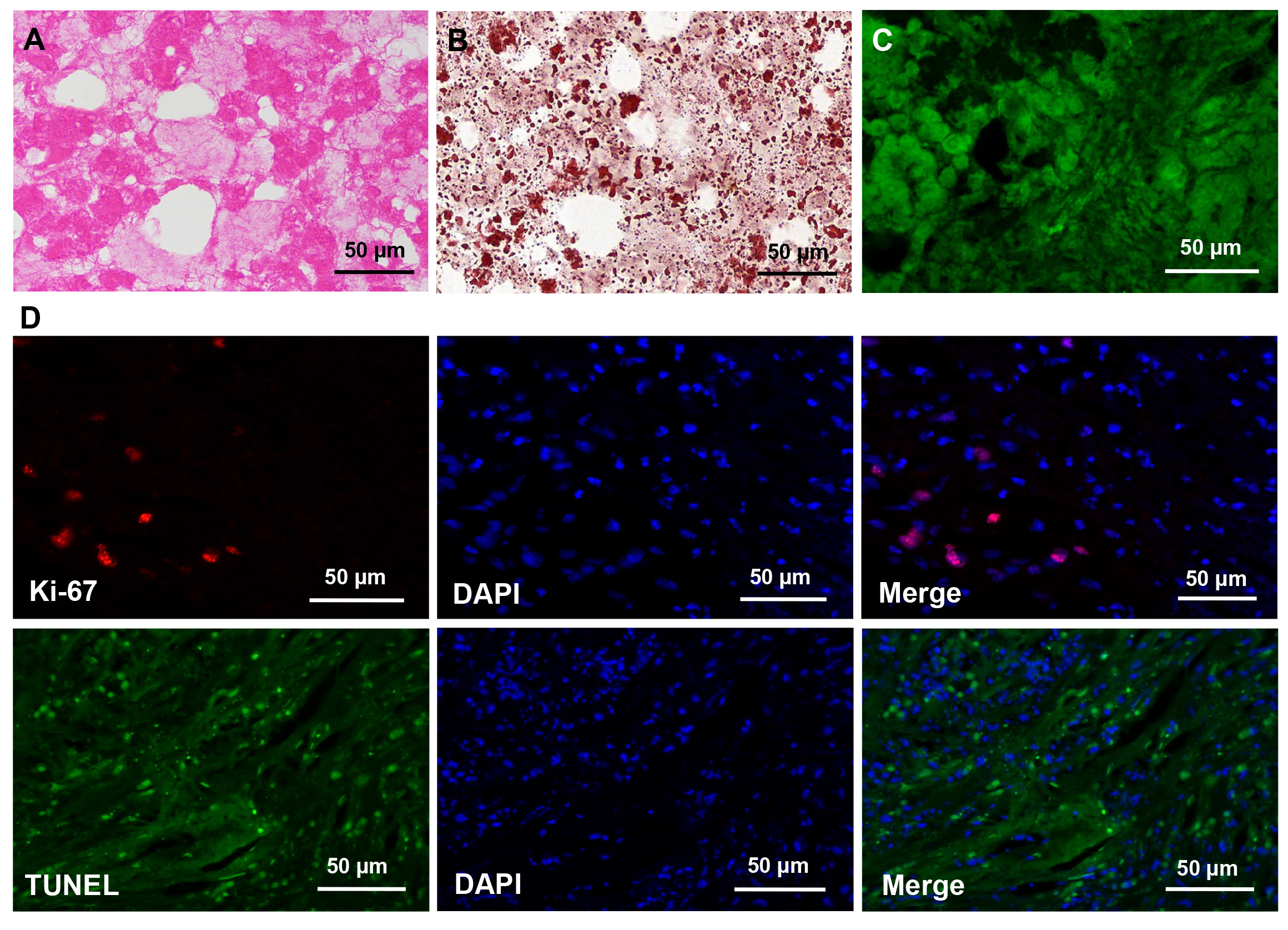


**Fig. S6 Histopathological examination. A-C** The H&E, oil red staining, and [fluorescence](javascript:;) [signal](javascript:;) of the primary tumor lesion in this patient. **D** The immunofluorescence histological analysis of the primary tumor lesion in this patient showed that there was a low expression rate of Ki-67, and a high expression rate of TUNEL.
