# Supplementary figures and images for: A clinical trial of super-stable homogeneous lipiodol-nanoICG formulation-guided precise fluorescent laparoscopic hepatocellular carcinoma resection
Source: J Nanobiotechnology. 2022 Jun 3;20:250. doi: 10.1186/s12951-022-01467-w (PMC9164554; doi:10.1186/s12951-022-01467-w)

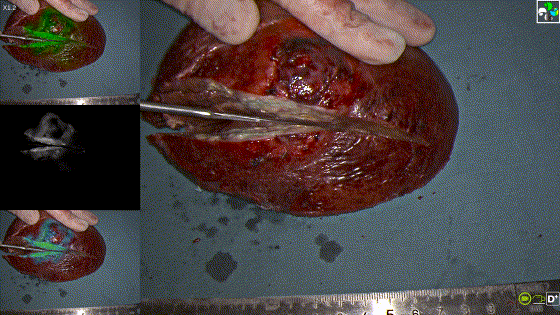

Supplement: Supplementary file 2 — Additional file 2: Vedio S1. Vedio of the fluorescent imaging effect when the tumor was dissected. [file 12951_2022_1467_MOESM2_ESM.gif]

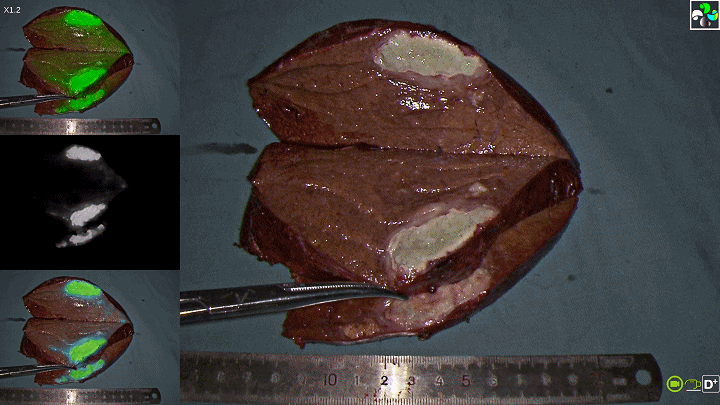

Supplement: Supplementary file 3 — Additional file 3: Vedio S2. Vedio of the fluorescent imaging effect when the tumor was dissected by layer. [file 12951_2022_1467_MOESM3_ESM.gif]
